# Preoperative medical assessment for adult spinal deformity surgery: a state-of-the-art review

**DOI:** 10.1007/s43390-023-00654-5

**Published:** 2023-02-22

**Authors:** Ayush Arora, Daniel D. Cummins, Aboubacar Wague, Joseph Mendelis, Rahul Samtani, Ian McNeill, Alekos A. Theologis, Praveen V. Mummaneni, Sigurd Berven

**Affiliations:** 1 Department of Orthopaedic Surgery, University of California - San Francisco UCSF, 500 Parnassus Ave, MUW320W, San Francisco, CA 4143-0728 USA; 2 Department of Neurological Surgery, University California, San Francisco, San Francisco, CA USA

**Keywords:** Deformity, Complications, Optimization, Risk factors, Frailty, Quality of care

## Abstract

**Introduction:**

The purpose of this study is to provide a state-of-the-art review regarding risk factors for perioperative complications in adult spinal deformity (ASD) surgery. The review includes levels of evidence for risk factors associated with complications in ASD surgery.

**Methods:**

Using the PubMed database, we searched for complications, risk factors, and adult spinal deformity. The included publications were assessed for level of evidence as described in clinical practice guidelines published by the North American Spine Society, with summary statements generated for each risk factor (Bono et al. in Spine J 9:1046–1051, 2009).

**Results:**

Frailty had good evidence (Grade A) as a risk for complications in ASD patients. Fair evidence (Grade B) was assigned for bone quality, smoking, hyperglycemia and diabetes, nutritional status, immunosuppression/steroid use, cardiovascular disease, pulmonary disease, and renal disease. Indeterminate evidence (Grade I) was assigned for pre-operative cognitive function, mental health, social support, and opioid utilization.

**Conclusions:**

Identification of risk factors for perioperative complications in ASD surgery is a priority for empowering informed choices for patients and surgeons and managing patient expectations. Risk factors with grade A and B evidence should be identified prior to elective surgery and modified to reduce the risk of perioperative complications.

## Introduction

Adult spinal deformity (ASD) has a significant impact on health-related quality of life (HRQOL). The incidence of ASD increases with age and may present in the form of scoliosis, kyphosis, spondylolisthesis, or rotatory subluxation, with accompanying neurological and functional deficits [2, 3]. Questionnaires that quantify HRQOL, including as the Owestry Disability Index (ODI), Scoliosis Research Society score (SRS), and Short Form 36 (SF-36), show that ASD patients have greater functional limitations, greater pain, and poorer mental health than age-matched controls and patients with other chronic medical conditions [3–6]. Generational decline of HRQOL is substantially higher in ASD patients compared to the general population [7]. Current estimates show that 27.5 million patients in the US are living with ASD, accounting for a significant societal burden that is projected to increase [8].

Surgical intervention for ASD is accompanied by a high incidence of complications (25–52%) [9, 10]. Major perioperative complications of ASD surgery, albeit less common, can include sepsis, adverse cardiac events, and respiratory and renal failure [11–14]. Both perioperative and late-stage (i.e. non-union) complications can reduce the chance of surgical benefit [15]. Moreover, the costs of ASD surgery can rise by up to 70% with re-admissions, establishing a financial incentive for reducing complications [16].

Knowledge of relevant risk factors for ASD surgery can empower clinicians and patients to gain insight into the expected postoperative course. Understanding which existing risk factors can contribute to complications may enable patients to make more informed choices preoperatively and allow for a better-shared decision-making ability [17]. Moreover, setting realistic expectations with patients prior to surgery can significantly improve patient satisfaction [18].

The purpose of this state-of-the-art review is to summarize and provide a level of evidence for, the association between proposed risk factors and perioperative complications following ASD surgery. The following risk factors will be explored: obesity, bone quality & osteoporosis, frailty, smoking, blood sugar, nutritional status, immunosuppression & steroid use, cardiovascular disease, pulmonary disease, renal disease, pre-cognitive function, mental health, social support, and opioid utilization.

## Methods

A literature search was conducted using the PubMed database, with intention of identifying publications pertaining to risk factors of interest and their relation to complications associated with ASD surgery. Keywords including the variables of interest (i.e. “obesity”, “BMI”, “frailty’” etc.) in addition to “adult spinal deformity” and “spine surgery” were used. In cases where the literature specific to ASD was limited, articles with cohorts of patients with spinal surgery beyond ASD were included. The included publications were assessed for level of evidence as described in clinical practice guidelines published by the North American Spine Society, reproduced below [1]:

A: Good evidence—Level I studies with consistent findings for or against recommending a variable as a risk factor.

B: Fair evidence – Level II or III studies with consistent findings for or against recommending a variable as a risk factor.

C: Poor evidence – Level IV or V studies for or against recommending a variable as a risk factor.

I: Insufficient or conflicting evidence not allowing a recommendation for or against intervention.

A summary statement for each specific risk factor was also generated.

## Results

### Obesity

Several studies have examined the effect of obesity on complications associated with surgical correction of ASD. A retrospective review by *Amin *et al*.* of 244 patients found that BMI > 35 was associated with significantly worse perioperative outcomes and higher cost compared to non-obese patients [19]. *Brown *et al*.* also found that obesity was associated with increased costs per quality-adjusted life year in patients undergoing ASD surgery [20]. However, other studies have reported no association between obesity and complications. A retrospective review by *Soroceanu *et al*.* of 448 patients found that BMI was not a significant risk factor for the development of post-operative medical complications [13]. These results were echoed by Elsamadicy et al. who reported that obesity did not significantly affect surgical outcomes, patient-reported pain scores, and 30-day readmission rates after complex spinal surgery [21]. *Yagi *et al*.* conducted a retrospective review of 195 patients who underwent surgical treatment of ASD with the aim of developing a predictive model for major complications at two years after surgery, and while several patient factors were found to be independent predictors of complications, obesity was not one of them [22].

In summary, obesity has been associated with increased complication rates and increased cost of care in spine surgery, but some studies analyzing the relationship between elevated BMI and surgical complications in adult spinal deformity surgery have shown a lack of significant association.

Grade of recommendation – I: There is insufficient evidence that obesity is a risk factor for perioperative complications in ASD surgery.

### Bone quality and osteoporosis

Bone quality is an important consideration when planning ASD surgery, and several reports in the literature underscore the value of bone mineral density (BMD) assessment as part of the preoperative medical workup. One retrospective review of 144 patients who underwent ASD surgery found that bone metabolism disorders are highly prevalent in elderly patients scheduled for spinal surgery and strongly recommended preoperative evaluation of BMD [23]. A recent retrospective comparative study found that the GAP score combined with BMI and BMD was better able to predict mechanical complications than the GAP score alone [24]. Another retrospective review of a multi-center ASD database evaluation by *Yagi *et al*.* concluded that low BMD was a significant risk factor for proximal junctional failure, recommending that surgeons consider prophylactic treatments when correcting ASD in patients with low BMD [25].

*Kuprys *et al*.* recommended that clinical practice guidelines be established on preoperative bone quality assessment for spine patients [26]. Bone quality assessment is typically done with a dual-energy x-ray absorptiometry (DEXA) scan, but it is important to note that in ASD, spine DEXA scans can be inaccurate. Use of DEXA of at least the hip and forearm was recommended for all ASD patients by *Gupta *et al*.*, as a single scan of the hip can lead to misdiagnosis, which can be costly in the setting of ASD [27].

Grade of recommendation – B: there is fair evidence that osteoporosis is a risk factor for perioperative complications in ASD surgery.

### Frailty

Frailty has garnered considerable attention as a potential metric that can be used to predict recovery and postoperative complications after ASD surgery. An international consensus group has defined frailty as, “A medical syndrome with multiple causes and contributors that is characterized by diminished strength, endurance, and reduced physiologic function that increases an individual’s vulnerability for developing increased dependency and/or death” [28]. With respect to ASD, studies have used the Adult Spinal Deformity Frailty Index (ASD-FI), the modified Frailty Index (mFI), and its truncated version, the mFI-5.

Each frailty risk score has been extensively validated. The ASD-FI stratifies patients as not frail (NF) (< 0.3 points), frail (0.3–0.5 points), or severely frail (SF) (> 0.5 points). Retrospective analyses of prospectively maintained multi-center databases have shown that greater patient frailty, as determined by the ASD-FI, is associated with a greater risk of serious complications (i.e. deep wound infection, pseudoarthrosis, proximal junctional kyphosis, wound dehiscence, reoperation) [29–31]. *Leven *et al*.* reported that mFI scores were also associated with increased rates of complications, reoperations, and mortality in a retrospective review of 1,001 ASD patients acquired from the National Surgical Quality Improvement Program multicenter database [32]. Finally, *Yagi *et al*.* utilized the mFI-5 to score patients as 0 (robust), 1 (prefail), or ≥ 2 (frail strata), and concluded that the mFI and the mFI-5 were similarly efficacious at predicting surgical adverse events (SAEs) (weighted Kappa: 0.87) [33]. Despite differing frailty index modalities, the literature agrees with its association with poor clinical outcomes and postoperative complications [34].

Grade of Recommendation – A: There is good evidence that frailty is a risk factor for perioperative complications in adult spinal deformity surgery.

### Smoking

The effects of tobacco smoke and ASD surgery are well documented. *Soroceanu *et al*.* and *Zhang *et al. reported that smoking was an independent risk factor for the development of post-operative medical complications, including cardiopulmonary and infectious [13, 35]. A history of smoking was found by *Puvanesarajah *et al*.* to be a risk factor for post-operative transfusions [36]. Using data from the SCOLI-RISK-1 Study, *Wilson *et al*.* found that a history of smoking significantly increased the risk of excessive intra-op bleeding [37]. Several studies have also found smoking history to be significantly associated with reoperations for ASD patients [38]. Smoking may also be an independent risk factor for mechanical failure, as shown in a series of 76 ASD patients by *Inoue *et al*.* [39]. In a study of 1,570 patients from the NSQIP database who underwent instrumentation > 7 levels for ASD, *Wade *et al*.* found that a history of smoking was an independent risk factor for surgical delay, and patients experiencing such delay demonstrated an almost sevenfold increase in mortality rate (3.4 vs. 0.5%) [40].

While several studies support smoking as a negative effector of outcomes and complication rates, some have found conflicting results. In a retrospective study*, Elsamadicy *et al*.* noted that a history of smoking did not affect 30-day readmission rates in 839 ASD patients [41]. While another study using data from the American College of Surgeon National Surgical Quality Improvement Program (ACS-NSQIP) database reported that smoking was not associated with higher 30-day complication rates or mortality, the authors recommended smoking cessation before spinal fusion given the known negative health effects of smoking.

Grade of Recommendation – B: There is fair evidence that smoking is a risk factor for perioperative complications in adult spinal deformity surgery.

### Blood sugar and diabetes

There is a positive association between diabetes and perioperative complications for patients with spinal surgery. Two retrospective reviews of the Healthcare Cost and Utilization Project Nationwide Readmissions Database (HCUP-NRD) have demonstrated diabetes as an independent risk factor for perioperative complications after controlling for other comorbidities [42, 43]. *Elasmadicy *et al*.* found that diabetic patients experienced increased readmission rates within 30 days and 90 days post-surgery, with infection being the most prevalent cause (30 days: 18.5%, 31–90 days: 7.4%) [42]. *Rubel *et al*.* determined that controlled and uncontrolled diabetes contributed significantly to increased odds of 90-day readmissions (top 3 causes: implant-related complications, disc herniation, postoperative infection) [43]. These findings are supported by a study by Guzman et al. using the Healthcare Cost and Utilization Project Nationwide Inpatient Sample (HCUP-NIS), where inpatient mortality in patients undergoing surgery for degenerative cervical spine pathology was shown to be associated with uncontrolled diabetes [44]. Only one study conducted by Shuman et al. found no association between complications and 90-day readmissions with diabetes, but the study reported an association between diabetes with prolonged length of stay and non-home discharge [45].

An unanswered question is whether the diabetic disease process or absolute blood glucose levels impact operative outcomes. Studies in which serum glucose has been directly measured have demonstrated its strength in predicting health outcomes. In patients without a formal diagnosis of diabetes, the risk of surgical site infection was doubled (2.1 to 4.4%) if perioperative blood glucose measurements were greater than 140 mg/dL (*p* = 0.01) [46]. Similar results were found in a case–control study by *Pennington *et al*.* in which multivariable analysis showed that history of diabetes mellitus, maximum daily glucose variation, and peak postoperative glucose were found to be significant predictors of wound infection [47]. These studies infer that both diabetes and elevated blood glucose levels increase patient risk for post-operative infection.

Grade of Recommendation – B: There is fair evidence that elevated blood sugar is a risk factor for perioperative complications in ASD surgery.

### Nutritional status

Several studies have linked malnutrition with post-surgical complications, but there is sparse literature on the association in the context of spine surgery. Serum albumin is widely used as a biomarker of nutritional status within patients undergoing surgical operations, with a value of less than 3.5 g/dL used as the standard marker of malnutrition [48]. Hypoalbuminemia was found to be independently associated with revision surgery for septic reasons and with acute postoperative infections within 30 days of aseptic revisions [49]. In a multivariate logistic regression analysis study of ASD patients, *Phan *et al*.* revealed low serum albumin to be an independent risk for 30-day mortality, length of stay greater than 5 days, all complications, pulmonary complications, renal complications, and intra-postoperative red blood cell transfusion [50]. Similar results were observed in a study by *Schonfeld *et al*.* in which serum albumin was associated with increased 30-day mortality, major complications, wound infections, and thromboembolic disease [51]. An additional study using the PearlDiver database by *Puvanesarajah *et al*.* found that patients with malnutrition had increased odds of 90 day major medical complications, including cerebrovascular accident, pulmonary embolism, pneumonia, myocardial infarction, and acute renal failure [52].

Grade of Recommendation – B: There is fair evidence that nutritional status is a risk factor for perioperative complications in ASD surgery.

### Immunosuppression and steroid use

There is a strong positive association between preoperative immunosuppression in patients with ASD and perioperative complications. Two retrospective reviews of the ACS-NSQIP database have shown this relationship for chronic oral steroid use when controlling for patient comorbidities and other potential confounds. *White *et al*.* found that oral and parenteral steroid use were associated with perioperative mortality, wound dehiscence, deep vein thrombosis, and blood transfusion [53]. *Di Capua *et al*.* showed chronic oral steroid use independently increased the risk of surgical site infection, urinary tract infection, or venous thromboembolism within 30 days of surgery [54]. A single-center study by *Tang *et al*.* also demonstrated steroid use for a chronic condition as a predictor of 90-day postoperative complications [55].

The effect of autoimmune disease, independent of immunosuppression, on perioperative complications is less certain. While a Nationwide Inpatient Sample analysis conducted by *Bernstein *et al*.* showed an increased risk of total perioperative complications for patients with rheumatoid arthritis (RA) compared to those without (29.9 vs. 24.6%, *p* < 0.001), it did not control for potential confounding factors, including steroid use, between patients with and without RA [56].

Grade of Recommendation – B: There is fair evidence that immunosuppression and steroid use are risk factors for perioperative complications in ASD surgery.

### Cardiovascular disease

The relationship between cardiovascular diseases and perioperative complications following surgery in patients with ASD is likely causal. A large cohort study of 30,330 patients who underwent spine surgery determined that risk factors for the development of cardiac complications within 30 days post-surgery included a history of cardiac disorders and treatments [57]. The development of a cardiac complication before discharge was also associated with a greater length of stay (7.9 versus 2.6 days; *p* < 0.001). Moreover, both *Zhao *et al*.* and *Soroceanu *et al. found hypertension to be a significant risk factor for postoperative deep venous thrombosis (DVT) [13, 58]. Utilizing registry data of 1,592 adult patients, *Guyot *et al*.* determined that previous cardiac history yielded statistically significant risk factors for cardiac complication after spine surgery, especially for patients in age groups 40–46 and > 65 [59].

Grade of Recommendation – B: There is fair evidence that cardiovascular disease is a risk factor for perioperative complications in ASD surgery.

### Pulmonary disease

There is an association between pulmonary diseases and perioperative complications following surgery in patients with ASD, with an emphasis on chronic obstructive pulmonary disease (COPD). In a retrospective study of 64,891 patients undergoing lumbar surgical procedures, *Murgai *et al*.* found that for patients with prior COPD, incidences of pulmonary collapse (5.6%; RR = 1.64), pneumonia (3.54%; RR = 2.63), and pleural effusion (2.55%; RR = 1.99) were significantly greater compared to patients without the risk factors [60]. *Tang *et al*.* also verified that postoperative complications were strongly associated with a history of severe COPD [55]. In a retrospective case–control study of 323 adult patients undergoing posterior spinal instrumentation and fusion for congenital scoliosis, *Wu *et al*.* found that the independent risk factors for the development of pulmonary complications included preoperative pulmonary disease [61]. Abnormal pulmonary function test (PFT) results have also yielded associations with pulmonary complications following surgery. In an analysis of 298 patients, Zhang et al. found that as pulmonary function decreased, pulmonary complication incidences increased: 31.6% [forced vital capacity (FVC) < 40%], 7.4% (FVC 40–60%) FVC, and 2.7% (FVC 60–80% FVC) [62].

However, other studies show no significant association between pulmonary risk factors and perioperative complications in ASD surgery. In a retrospective, multi-center, case–control study of 953 ASD patients, *Schwab *et al*.* found that cardiopulmonary status and co-morbidities were not significantly associated with major perioperative complications [63]. *Liang *et al*.* also found a similar result in a retrospective clinical study of 88 patients with moderate or severe pulmonary dysfunction, indicating that prior respiratory symptoms and FVC play little role in postoperative pulmonary complications [64].

Grade of Recommendation – B: There is fair evidence that pulmonary disease is a risk factor for perioperative complications in ASD surgery.

### Renal disease

Multiple studies suggested that patients with chronic kidney disease (CKD) (Stage 3–5) have higher complication rates and increased mortality following adult spinal surgery. A study in 2017 by *Bains *et al*.* demonstrated that patients with CKD had a 1.78 odds ratio (OR) of increased mortality following spine surgery compared to patients with normal kidney function [65]. If the patient was on dialysis, that OR increased to 4.18. Another study by *Adogwa *et al*.* noted that patients with CKD were more likely to be admitted to the ICU (53 vs 29%), have post-operative delirium (27.8 vs 8.4%), have UTIs (27.8 vs 6.9%), and have DVTs (5.6 vs 0.4%) when compared to those without CKD [66]. A 2012 study by *Chikuda *et al*.* found that there was a ten-fold increase in-hospital mortality in patients who were undergoing dialysis compared to patients who were not undergoing dialysis [67].

Grade of Recommendation – B: There is fair evidence that renal disease is a risk factor for perioperative complications in ASD surgery.

### Pre-operative cognitive function

Numerous studies suggest that poor preoperative cognition may be a significant risk factor for post-operative delirium. The prevalence of poor preoperative cognition may be high, with *Lee *et al*.* finding 38% of patients undergoing lumbar spine surgery to meet diagnosis of cognitive impairment using a Mini-Mental State Exam (MMSE) [68]. In a study by *Toombs *et al*.* patients with preoperative dementia and psychotic disorders had more complications than controls in patients undergoing surgery for ASD [69]. *Adogwa *et al*.* noted cognitive impairment to be a risk factor for postoperative delirium and concluded that cognitive impairment assessments should be considered in the preoperative evaluation of elderly patients prior to surgery [70]. Although many studies discuss post-operative delirium as a common consequence of preoperative cognitive decline, longer-term outcomes are not conclusive and deserve further investigation.

Grade of Recommendation – I: There is insufficient evidence that pre-operative cognitive function is a risk factor for perioperative complications in ASD surgery.

### Mental health

A likely non-causal association has been demonstrated between mental health factors and perioperative complications following surgery in ASD patients. The strongest evidence for this was provided by *Shah *et al*.* who noted that depression and anxiety were associated with increased rates of perioperative infection and respiratory complications following surgical correction of ASD after matching patients for sex, age, and Charlson Comorbidity Index (CCI) [71]. While *Soroceanu *et al*.* also found an increased rate of medical complications for patients with depression on univariate analysis (RR = 1.6, *p* = 0.0001), the result did not hold on multivariate analysis [13]. Using the SRS-22r, *Watanabe *et al*.* showed that unfavorable mental health scores were associated with perioperative infection, hematoma, DVT, and delirium [72]. These results did not control for potential confounding variables, including comorbidities or preoperative disability, which were greater in patients with worse mental health. Similarly, a NIS analysis performed by *Toombs *et al*.* showed increased rates of major complications in the perioperative period for patients with psychotic disorders, but the study also failed to control for potential confounding factors [69]. Finally, many clinical studies have failed to find any relationship between perioperative complications following ASD surgery using metrics such as a preoperative diagnosis of depression, Koenig Depression Scale (KDS), and quantitative scale of psychiatric distress [73–75]. Therefore, the most available literature is inconclusive and fails to control for other confounding factors present in patients with mental health illnesses.

Grade of Recommendation – I: There is insufficient evidence that mental health is a risk factor for perioperative complications in ASD surgery.

### Social support

Social support is the presence of people and community institutions that can provide supportive behaviors and interventions to help reduce the stress and burden associated with major life events. There is a paucity of data published regarding the impact of social support on outcomes in patients undergoing surgery for ASD. Literature in cardiac surgery and orthopaedic surgery suggest social support may be an important predictive factor of surgical outcomes [76]. For example, *Adogwa *et al*.* found that one year after surgery, single patients were observed to have a longer length of stay compared to married patients in 430 patients who underwent elective spine surgery [77]. However, no differences in complications, 30-day readmissions, or patient-reported outcomes were found. To date there have not been any studies evaluating social support in the ASD population, which may be a consequence of the inability to effectively assess social support in large-scale databases and registries [8].

Grade of Recommendation – I: There is insufficient evidence that poor social support is a risk factor for perioperative complications in ASD surgery.

### Opioid utilization

There is sparse literature specifically addressing pre-operative opioid use and perioperative complications for ASD. A multicenter retrospective review by *Raad *et al*.* found daily preoperative narcotic use independently predicted prolonged hospital length of stay and longer ICU stay [78]. One single-center review by *Mesfin *et al*.* found no significant difference in major and minor complications between patients taking opioids and those taking non-opioid analgesics preoperatively [79]. *Elsamadicy *et al*.* also found no significant difference in complications between preoperative narcotic users and non-narcotic users for patients receiving five or more level fusion for ASD [80]. Other potentially relevant variables for risk of perioperative complications, such as duration or dosage of opioid utilization, have yet to be assessed.

Grade of Recommendation – I: There is insufficient evidence that preoperative opioid use is a risk factor for perioperative complications in ASD surgery.

## Discussion

Table 1 summarizes that patient frailty had the highest grade of evidence for perioperative complications. There is also fair evidence for bone quality, smoking, high blood sugar & diabetes, nutritional status, immunosuppression/steroid use, cardiovascular disease, pulmonary disease, and renal disease as independent risk factors for complications following operative management of ASD.

**Table 1:** Summarized grade of recommendation for perioperative complications by risk factor

Grade of recommendation	Risk factor	Key reference(s)
A: Good evidence as a risk factor	Frailty	Leven et al. 2016 [32]; Yagi et al. 2019 [33]
B: Fair evidence as a risk factor	Bone quality and osteoporosis	Yagi et al. 2019 [34]
	Smoking	Soroceanu et al. 2016 [13]; Zhang et al. 2018 [35]
	Blood sugar control	Cho et al. 2012 [81]
	Nutritional status	Phan et al. 2018 [50]
	Immunosuppression and steroid use	White et al. 2019 [53]; Di Capua et al. 2017 [54]
	Cardiovascular disease	Soroceanu et al. 2016 [13]
	Pulmonary disease	Zhang et al. 2004 [62]; Liang et al. 2010 [64]
	Renal disease	Adogwa et al. 2018 [66]
I: Indeterminate evidence as a risk factor	Pre-operative cognitive function	Adogwa et al. 2018 [70]
	Mental health	Shah et al. 2019 [71]
	Social support	Adogwa et al. 2017 [77]
	Opioid utilization	Elsamadicy et al. [80]

The strength of evidence for frailty’s association with complications has already been applied clinically. Numerous applications currently utilize frailty as a preoperative risk assessment tool to predict postoperative complications and death for elderly surgical patients outside of spine surgery [81, 82]. Frailty has been used to predict discharge time and mortality after laparoscopic colorectal and liver surgery, with higher scores predicting increases in complications, mortality, and extended length of hospital stay [83, 84]. Similarly, the ASD-FI, mFI, and mFI-5 have been utilized to predict complications in ASD surgery [32, 33]. It is possible that certain frailty indices perform better in predicting perioperative complications. As shown by *Burn *et al*.* frailty indexes are typically calculated by summing the number of functional deficits for a specific patient divided by the total number of possible deficits [85]. Those that utilize more deficits to facilitate the calculation could potentially yield better measures but also require greater resources to acquire. Additional studies comparing the predictive validity of different frailty indexes could produce information useful to inform preoperative guidance.

Variables assigned a *Grade B: Fair Level of Evidence* consisted of most literature supporting an association with perioperative complications, with some variation. For example, while the preponderance of literature suggests that impaired bone quality and bone metabolism compromise surgical outcomes, preoperative bone quality assessment can differ between males and females, and DEXA scans can be inaccurate depending on the scanned region [86, 87]. Future studies that control for patient gender and region of scanning would be of further benefit. A history of smoking, cardiovascular disease, and respiratory disease also presented fair evidence as risk factors for post-operative complications, accompanied by a minority that found conflicting results. Such studies, one being the case–control series by *Schwab *et al*.* that failed to find an association between cardiovascular disease and perioperative complications, had less than 10% of patients with complications, citing a necessity for improved matching of additional risk factors [63].

Moreover, smoking, cardiovascular disease, and respiratory disease may predispose patients to long-term complications that are not captured by most available reports. For example, certain studies report that smoking cessation before spinal surgery may improve short-term clinical outcomes, while others report smoking as an independent risk factor for longer-term complications (implant failure, pseudoarthrosis, revisions) [41, 88]. Preoperative pulmonary disease had similar associations with longer-term complications (reoperation) and short-term complications (respiratory failure, prolonged mechanical ventilation) [61]. A single study may be unable to capture the immediate and long-term complications due to certain risk factors, but the literature suggests a trend that such risk factors are indeed associated with either short-term of long-term post-operative complications.

Patient management of risk factors is also an important consideration. For example, it has been generally accepted that uncontrolled high blood sugar predisposes patients to increased cardiovascular, infectious, and additional pathophysiologic diseases [89–91]. The studies found in this literature search tended to establish uncontrolled diabetes as an independent risk factor for complications and mortality, while the risks from controlled diabetes ranged from having no association to significant ones with increased infection risk and length of stay. Uncontrolled and controlled diabetes may have different risk profiles, and two patients presenting with diabetes might be predisposed to different complications depending on the disease management. In addition, blood sugar level and diabetes should not be interpreted as binary variables such as “high blood sugar”, “low blood sugar”, “diabetic”, or “not diabetic” when evaluating patients for risk factors. Two of the studies referenced found that doubling blood glucose measurements and increasing daily glucose variation were significant predictors of surgical infections [47, 48]. Measurement of Hemoglobin A1c may present a better method to evaluate the effects of blood sugar on perioperative complications, but few studies have done so in the setting of ASD surgery. In a similar line, the association between renal disease and complications was found to be well-outlined, especially for patients with end-stage renal disease who are dependent on dialysis. However, few studies determined perioperative complications for patients earlier in the disease (i.e. CKD stage 1–3). Future reports on comparisons between patients with preserved eGFR in early-stage CKD and those on dialysis or late-stage CKD could provide additional recommendations.

Few studies discussed the relationship between nutritional status and perioperative complications in ASD surgery. Nevertheless, the fact that low albumin has been associated with infections, vascular complications, and organ dysfunction is in concordance with immune dysregulation from malnutrition [92]. Utilizing prealbumin may be a more accurate biomarker than albumin for nutritional status, given prealbumin’s shorter half-life and reflection of the patient’s most recent nutritional state [93].

The effect of steroid usage on perioperative complications makes intuitive sense but requires additional inquiry. Steroid usage was reported to be significantly associated with perioperative complications in the referenced studies. Large-database studies (> 500,000 patients) reported significantly increased risks of superficial surgical site infections (SSI), deep SSIs, and mortality (4x) with steroid use [94]. Yet, few studies distinguished between complication rates based on the type of steroids and between those who were chronic or intermittent users. Variations in steroid dose may also contribute to complications, as dose can correspond to different levels of immunosuppressive effects [95]. Overall, the literature is convincing that immune dysregulation secondary to steroid use and malnutrition is associated with perioperative surgical complications.

Insufficient evidence was available to link the following variables to complications following ASD operations: obesity, mental health, social support, cognitive function, and opiate use. For risk factors that had significant relationships to complications, such studies failed to control for potential other risk factors. Such variables, however, may still be associated with other predictors of perioperative complications. For example, while obesity, mental health, and social support had insufficient evidence, all three variables are implicated as independent risk factors for cardiovascular disease and can occupy a spectrum that produces varying effects on perioperative complications [96–98]. Obesity is typically categorized into Class I (30 < BMI < 35), Class II (35 < BMI < 40), and Class III (BMI > 40), and the rate of complications may increase with a higher class of obesity [99]. A retrospective clinical study conducted by *Yokoo *et al*.* determined that BMI > 30 was a risk for blood transfusions, while BMI > 35 increased the risks for unplanned intubation and cardiac events [100]. Clearly, risk profiles can change and become more pronounced with a higher extent of disease. As such, many clinicians use the variables having insufficient evidence for preoperative assessment. For example, *Keller *et al*.* showed that patients afflicted with obesity can necessitate adjustments to equipment, altered medication delivery, increased cardiac monitoring, and additional focus on oxygenation and wound healing [99]. A possible reason that certain risk factors had insufficient evidence is that most studies did not stratify BMI, mental health, and social support into sufficient ranges.

The results found by this review should inform the risk factors that should be taken into consideration during the preoperative management of patients undergoing ASD surgery. With knowledge of patient frailty, in conjunction with factors that have fair evidence of risk (cardiovascular, renal, and pulmonary diseases), clinicians may facilitate better-shared decision-making ability regarding the appropriateness of surgery and expected outcomes. Risk factors may be modified to optimize patient health prior to surgery and reduce the chance of complications. Standard Work protocols incorporating modifiable risk factors for deformity patients have been suggested by *Burton *et al*. and Sethi *et al*.* in extensive meta-analysis [101, 102]. Finally, knowledge of risk factors that contribute to complications may help set patient expectations preoperatively.

### Applications of Pre-operative medical assessment in quality assessment

The ability of clinicians to stratify patient risk is important for the setting of benchmarks on expected complications [103]. Patients with higher frailty index scores, more medical comorbidities, and greater surgical invasiveness may be more likely to incur complications than those without underlying risk factors. While higher complication rates in the former groups may be expected, equivalent complication rates in the latter group may represent gaps in care. It was vital to understand whether those who incur a complication are high-risk or low-risk. Patients with greater medical comorbidities would have a higher expected benchmark for complications, while patients with fewer risk factors would have a lower expected benchmark. Therefore, the assessment of postoperative outcomes in ASD patients without evaluation of individual patient risk profiles could result in incorrect assumptions about quality of care received.

### Limitations

One key limitation was the limited granularity of data available in the literature that could enable the stratification of patients based on specific diagnosis and surgical procedures. In most studies, cohorts consisted of patients with a variety of spinal deformities, with little distinction between types of deformity. Depending on the diagnosis, a different set of risk factors could be less or more predictive of post-operative complications. Due to limitations in both quality and volume of studies pertaining to ASD complications, it was difficult to obtain studies that could unanimously focus on patients with a singular set of ASD diagnosis, procedures, or surgical approaches. While the risk factors assigned a grading of good and fair evidence in this review may be applicable broadly to ASD patients, the applicability may vary depending on the specific patient pathology.

Additionally, certain studies lacked a reliable sample size, some having so few patients with complications that an adequate determination of the association could not be made. Others failed to control for the patients’ full risk profiles. Most importantly, for certain risk factors, including social support and opioid utilization prior to surgery, a limited number of studies discussed the association with complications specifically for ASD patients. While social support and opioid utilization have been identified as risk factors for complications following cardiovascular and shoulder surgery, extrapolating such relationships to ASD is difficult and future studies are needed [104, 105].

Finally, a variety of studies discussed risk factors in the context of long-term complications, such as reoperation and revision surgery [38, 106]. However, such studies may have underestimated complication rates by failing to utilize an appreciable window of time between a patient’s primary surgery and reoperation. As reported by *Ahmed *et al., revisions for scoliosis can continue past 2-years after the index operation, with a 5-year survivorship of 93.9% [107]. Complications and associated preoperative risk factors are likely underreported in studies with only 2 years of follow-up.

### Future directions

Future research focusing on risk factors deemed to have an insufficient level of evidence in association with postoperative complications is needed. Upcoming studies should stratify patients by diagnosis, procedure, and procedural approach (ex. staging, anterior, posterior) in efforts to control for confounding operative variables and focus on preoperative risk factors. Additionally, risk factors that include social support have shown good promise in predicting outcomes following surgery, and future studies could utilize questionnaires [i.e. Risk Assessment and Prediction Tool (RAPT) score] that factor in metrics of community support and in-home assistance [108]. Complications should be classified as peri-operatively, short-term (at 30 days, 90 days, and 1 year post-operatively), and long-term (at 5 years and beyond) to capture revisions, reoperations, and pseudoarthrosis. Classification systems such as the Clavien-Dindo system could divide complications into classes ranging from lower severity (requiring pharmacological intervention) to high severity (morbidity) [109]. The standardization of complications into discrete classes could allow for more comparative studies in the future.

### Implications for the development of predictive models

The primary goal of this report was to identify preoperative risk factors that were associated with complications following surgery for ASD. The next step would be to develop predictive models that incorporate and appropriately weigh the pertinent risk factors identified in this study. So far, currently utilized indices and risk scores have variable predictive utility. Specifically, *Ondeck *et al*.* demonstrated that numerically tabulated mFI indices and CCI were as predictive of complications after posterior lumbar fusion surgery as the easily obtained demographics and American Society of Anesthesiology (ASA) score [110]. *Deogaonkar *et al*.* and *Hartin *et al*.* demonstrated that the Fusion Risk Score (FRS), comprised of weighted demographics, comorbidities, surgical approach, levels, and osteotomies, also had a significant association with postoperative medical complications [111, 112]. Evidently, existing risk scores including the mFI and FRS hold promise but require additional validation along with other components of the patient’s risk profile. Future work should ideally utilize machine learning develop to predictive models that incorporate the risk factors identified in this study along with validated risk indices. Development of machine learning models would require large cohorts of patients with granular information in efforts to derive data-driven tools. Such predictive tools would serve to streamline the risk assessment process and enable rapid generation of expected patient outcomes. The relative contribution of each component of the model could be optimized as more patient information becomes available.

## Conclusion

Comprehensive preoperative medical assessment decision-making is an important process that involves weighing the risk and benefits of surgery for ASD. The goal of this state-of-the-art review was to summarize the level of evidence for proposed risk factors for perioperative complications following ASD surgery. The included publications were assessed for level of evidence, as previously described in clinical practice guidelines published by the North American Spine Society, and a summary statement was generated using information synthesized from a review of all publications pertaining to each particular risk factor. Of the risk factors identified, frailty had good evidence as an independent risk factor for perioperative complications, while smoking, hyperglycemia, nutritional status, renal disease, cardiovascular disease, pulmonary disease, bone quality, and immunosuppression/steroid use had a fair level of evidence. Insufficient evidence was yielded for obesity, mental health, cognitive function, social support, and opioid use. Overall, we recommend using patient frailty in conjunction with cardiovascular, renal, and pulmonary diseases along with other factors that have fair evidence of risk. Clinicians may utilize important risk factors identified in this study to conduct shared decision-making regarding the appropriateness of spine surgery, to set patient expectations, and to guide preoperative planning.

## Data Availability

The data used to support the findings of this study are available from the corresponding author upon request.
